# Immunomodulatory function of cannabinoid receptor 2 and its agonist osteogenic growth peptide in health and cancer: a study in mice and humans

**DOI:** 10.1038/s41388-025-03399-9

**Published:** 2025-04-30

**Authors:** Jennifer Ana Iden, Nathalie Ben-Califa, Aaron Naim, Tamar Liron, Drorit Neumann, Yankel Gabet

**Affiliations:** 1 Department of Anatomy and Anthropology, Faculty of Medical and Health Sciences, Tel Aviv University, Tel Aviv, Israel; 2 Department of Cell and Developmental Biology, Faculty of Medical and Health Sciences, Tel Aviv University, Tel Aviv, Israel

**Keywords:** Colorectal cancer, Cancer genomics

## Abstract

Colon carcinoma is among the most prevalent malignant tumors, with inflammation being the primary risk factor. Cannabinoid receptor 2 (CB2/CNR2) has complex immunomodulatory functions. Therefore, we investigated the role of osteogenic growth peptide (OGP), an endogenous selective CB2 agonist, in colon carcinogenesis and immune modulation in transgenic mice (Apc^Min/+^).We injected 8-week-old (progression phase) or five-week-old (initiation phase) Apc^Min/+^ mice with OGP or vehicle weekly for 8 weeks or 4 weeks, respectively. During the progression phase, OGP-treated mice displayed significantly fewer tumors in the large intestine and smaller tumors in the small intestine. During the initiation phase, OGP significantly attenuated adenomagenesis in both the small and large intestine, decreased IL-6 and IL-4 levels, increased splenic anti-tumor CD8+ T cells, and diminished populations of tumor-promoting myeloid-derived suppressor cells. Further, we used exomic analyses of UKBiobank patients to determine the relationship between *CNR2* polymorphisms and tumor-associated myeloid cells in humans. We found that the common CNR2-Q63R polymorphism is associated with monocyte count. Our results suggest that CB2 activation via OGP attenuates tumorigenesis and adenoma growth by modulating immune cells, corroborated by a significant association between *CNR2* polymorphisms and monocytopoiesis in humans.

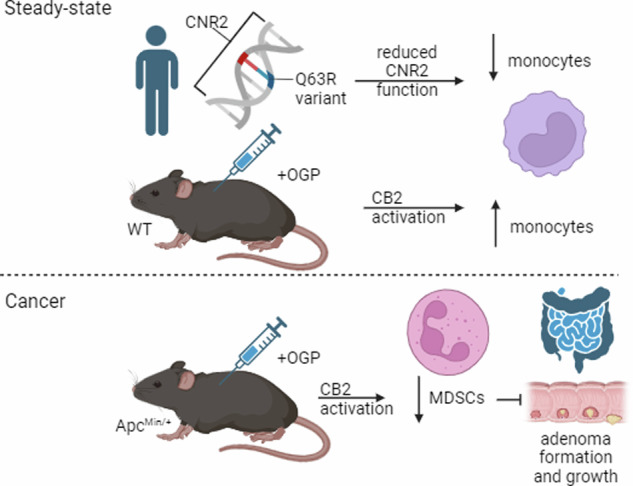

## Introduction

Colon cancer is one of the most prevalent malignancies and remains a significant cause of cancer-related mortality [1]. Chronic inflammation of the inner lining of the intestine is a major risk factor for colon carcinogenesis [2]. An emerging therapeutic target is the endocannabinoid system, comprising endogenous lipids, enzymes, and receptors, including cannabinoid receptor 2 (CB2) [3–5]. CB2 has been shown to play a role in various biological processes, including immune response, pain modulation, and, notably, tumorigenesis [6, 7]. We have previously shown that lack of endogenous CB2 activation ameliorates colorectal cancer (CRC) and non-melanoma skin cancer [8, 9]. Osteogenic Growth Peptide (OGP), an endogenous peptide with pleiotropic effects, including bone formation and anti-inflammatory action, has recently been identified as a CB2 selective agonist [10].

OGP(1-14) is a 14-amino acid peptide that is present in the nano-micromolar range in human serum, and is a well-established bone anabolic agent [11]. It is complexed to α2-macroglobulin, and after dissociation, is proteolytically cleaved into the pentapeptide OGP (10-14), hereafter referred to as OGP [11]. Pertinently, OGP has also been shown to regulate myelopoiesis in mouse models of myeloid suppression [12].

Myeloid-derived suppressor cells (MDSCs) play a critical role in the immune microenvironment of CRC [13, 14]. These immature myeloid cells contribute to tumor immune evasion and progression by suppressing T cell responses [15–17]. MDSC activity is known to be regulated by interleukin-4 (IL-4) and interleukin-6 (IL-6) [18–20]. IL-6 is pro-inflammatory cytokine involved in various cellular processes, including immune responses and tumor progression [20–22]. In colon cancer, IL-4 and IL-6 promote tumor growth and metastasis. They contribute to the expansion, survival, and suppressive function of MDSCs [21, 23]. Additionally, there is evidence that CB2 activation downregulates IL-6 expression and suppresses MDSC activity in mouse models of CRC [8, 24]. Apc^Min/+^ mice have been widely used to study the tumorigenic effects of MDSCs [25–27].

The Apc^Min/+^ mouse model is a valuable genetic model for studying hereditary colon cancer as it presents in humans [28]. These mice carry a mutation in the tumor-suppressor gene, *Adenomatous polyposis coli* (*APC*), which predisposes them to spontaneous development of multiple intestinal adenomas [29]. Furthermore, these mice develop severe macrocytic anemia accompanied by splenomegaly due to ineffective erythropoiesis, wherein the typical differentiation of erythroid precursors is blocked in early stages [30]. In addition to hematologic disorders, this model is particularly useful for studying multiple phases of cancer development as juvenile Apc^Min/+^ mice do not have adenomas: there is a 5-week delay in adenoma development during which a sufficient amount of intestinal cells accumulate the mutation [31]. The formation of adenomas from week five to eight is exponential, after which the adenomas continue to form, although at a much slower rate, with substantial growth in size from week eight to twelve [31, 32]. Therefore, we used two distinct paradigms to investigate the effect of OGP administration on adenoma formation (initiation) and growth (progression) in Apc^Min/+^ mice.

At a genome-wide level, the *CNR2* gene has been linked to bone mineral density, osteoporosis, CRC, and severe acute respiratory syndrome from COVID-19 [8, 33–37]. Additionally, *CNR2* variants have been linked to peripheral immune cells, particularly eosinophils and lymphocytes [38, 39]. The CNR2-Q63R polymorphism, a glutamine (Q)/ arginine (R) substitution at codon 63 of the *CNR2* gene, alters the polarization state of the receptor and influences its responses to cannabinoids and affects endocannabinoid-dependent lymphocyte proliferation [40–42]. We previously showed that a contributing single nucleotide polymorphism (SNP) to Q63R, rs2501432, is potentially linked to colon cancer [8]. Other groups have specified the risk of specific and common *CNR2* variants, mainly Q63R, in ulcerative colitis, Crohn’s disease, thrombocytopenia purpura, rheumatoid arthritis, and diabetic kidney disease [42–48]. Due to the apparent dual role of *CNR2* on the genetic level to impact immune cell populations and disease pathogenesis, we assessed exome-wide association studies and gene-based burden tests for a link between *CNR2* variants and peripheral monocyte count.

## Materials and methods

### Mice

Mice on a C57Bl/6J genetic background were bred in the specific pathogen-free (SPF) animal facility in Tel Aviv University. All mice were housed as per IACUC guidelines in temperature-controlled rooms with a 12-h light cycle and were given water and pelleted chow *ad libitum*. All experiments were conducted in accordance with the guidelines and with the approval of the Tel Aviv University Animal Care and Use Committee (Protocols 01-18-059, 01-18-060, and 01-21-010). At least six mice were used per group. For steady-state analyses, 6-week-old female WT mice were injected intraperitoneally with either 700 ng OGP (synthesized at Tel Aviv University) or vehicle control (saline) on day 0, 7 and 14, and sacrificed on day 15. Apc^Min/+^ mice were obtained from the Jackson Laboratory and subsequently bred in-house using heterozygous males on a C57BL/6J background. For progression phase studies, 8-week-old male and female Apc^Min/+^ mice were injected with 100 ng OGP daily, 700 ng OGP weekly, or a vehicle control for 8 weeks. For initiation phase studies, 5-week-old male and female Apc^Min/+^ mice were injected once weekly with 700 ng OGP for 4 weeks.

### Cell culture

CT26 (murine colon carcinoma) cells were grown in T75 flasks in RPMI supplemented with 10% FBS and 1%. penicillin/streptomycin and were incubated at 37 °C in a humidified 5% CO_2_ incubator. The cells were received from Dr. Anat Globerson Levin of Tel Aviv Sourasky Medical Center. For collection of CT26 conditioned media, cells were grown to 80% confluency and the supernatant was collected, filtered through a 0.22-micron syringe filter (Millex®- GV filter unit, Merck, Herzilya, Israel) and used immediately or stored at -20 °C. To assess cell proliferation, CT26 cells (2 × 10^4^ cells/well) were seeded in a 96-well plate and incubated overnight (18 h). The cells were then starved in serum-free media for 2 hr followed by OGP treatment (10^−9^, 10^−10^, or 10^−^^12^ M) or vehicle control for 1 h before the addition of 10% FBS. After 24 or 48 h, MTT solution was added for 4 h. After dissolving the formazan crystals in DMSO, the absorbance was measured at 560 nm on a spectrophotometer (SpectraMax I3X, San Jose, California, USA). For mRNA analysis, CT26 cells were seeded at 10^6^ cells/well in a 6-well plate overnight. Cells were starved for 2 h, in serum-free media, followed by the addition of OGP (10^−9^, 10^−10^, or 10^−12^ M). After 4 h, cells were collected for RNA extraction.

### MDSC generation in vitro

MDSCs were generated as described in Dufait et al. with minimal modifications [49]. Briefly, bone marrow was harvested from the tibia and femur of 8-week-old male mice and cultured in RPMI supplemented with 10% FBS and 1%. penicillin/streptomycin for 4 h. Cells remaining in suspension were washed, seeded in a 12-well plate, and starved for 2 h in serum-free RPMI. Cells were treated with OGP 10^−12^ M or vehicle control for 1 h before adjusting the media to 20% RPMI, 10% conditioned media from L929 cells, and 70% conditioned media from CT26 cells (media cocktail was supplemented with 10% FBS and 1% penicillin/streptomycin), After 72 h, cells were collected for flow cytometry or maintained for nitric oxide assessment.

### Nitric oxide assessment

After 72 h of differentiation in the media cocktail (see above), primary murine bone marrow cells were washed with PBS and starved for 2 h in serum-free RPMI. Cells were treated with OGP 10^−12 ^M for 1 h, followed by the addition of 100 ng/mL LPS and the media cocktail. After 48 h, the nitric oxide in the supernatant was measured by the Griess Reagent System (Promega, Madison, WI, USA) according to the manufacturer’s instructions.

### Genotyping

Genomic DNA was extracted from 2 mm tail clippings using Extracta DNA Prep for PCR (Quantabio, Beverly, MA). PCR was performed with DreamTaq Green PCR master mix (Thermo Scientific, Waltham, MA, USA). The following primers were used: Apc^Min/+^ wild-type forward, 5′-GCCATCCCTTCACGTTAG-3′, Apc^Min/+^forward, 5′-TTCTGAGAAAGACAGAAGTTA-3′, and Apc^Min/+^ common antisense, 5′-TTCCACTTTGGCATAAGGC-3′ for Apc^Min/+^.

### RNA extraction and qPCR

Total RNA was extracted from CT26 cells using TRIzol reagent (Invitrogen, Carlsbad, CA, USA), and qPCR was performed using cDNA generated from 1 µg of total RNA with a cDNA synthesis kit (Quantabio, Beverly, MA, USA). qPCR reactions were carried out on 20 ng cDNA per reaction using SYBR Green PCR master mix (Quantabio, Beverly, MA, USA). Reactions were carried out on a Step-One (Thermo Fisher, Waltham, MA, USA) analysis system. Relative expression values were quantitated using the comparative cycle threshold method and normalized to mouse β-actin. The following primer sequences (5′-3′) were used:

**Table:** 

β-actin_F	GTCACCCACACTGTGCCCATC
β-actin_R	CCGTCAGGCAGCTCATAGCTC
IL-6_F	CCGGAGAGGAGACTTCACAG
IL-6_R	GGAAATTGGGGTAGGAAGGA

### Flow cytometry

Single-cell suspensions were obtained by manually homogenizing harvested spleens in a petri dish or flushing the marrow contents from tibia with a 27 G needle. To isolate cells from the epithelial layer of colons, the large intestines were harvested, flushed with a blunt needle, cut longitudinally then into 1 mm fragments, and incubated at 37 °C in PBS with 10 mM EDTA and 10% FBS for two cycles of 45 min, collecting the supernatant after each cycle. Red blood cells were lysed using ACK Lysis buffer (Life Technologies, Carlsbad, CA USA) where necessary. Cells were washed with PBS supplemented with 2 mM EDTA and 5% FBS, filtered through a 70 μm cell strainer (Falcon, Corning Incorporated), and then resuspended. T cells were stained with anti-CD3-FITC, anti-CD4-PE and anti-CD8-APC for 30 min on ice, while myeloid cells were stained with anti-CD45-Pacific Blue, anti-CD11b-PE-Cy7, anti-CD11c-PE, anti-Ly6G-FITC, anti-Ly6C-PerCP-Cy5.5, and anti-Siglec-F-APC or anti-F4/80-APC for 45 minutes on ice. For erythroid cells, bone marrow cells were stained with anti-CD71-PE and anti-Ter119-APC for 20 min on ice. For MDSC polarization experiments, cells were stained with anti-CD45-BV605, anti-CD11b-PE, anti-Ly6G-Pe-Cy7, and anti-Ly6C-BV421. For the epithelial layer of colons, cells were stained with anti-CB2 (Cayman Chemical, Ann Arbor, MI, USA) and HyLite™ Fluor 647 (Anaspec, Fremont, CA, USA), anti-CD45-BV605, and anti-CD11b-PE. All antibodies were purchased from BioLegend (San Diego, CA, USA) unless indicated otherwise. After staining, cells were washed twice with PBS and the fluorescence was assessed with a CytoFlex5L (Beckman Coulter, Brea, CA, USA). Dendritic cells were classified as CD45 + CD11b + CD11c + , macrophages as CD45 + CD11b + F4/80+, eosinophils as CD45 + CD11b+Siglec-F+, PMN-MDSCs as CD45 + CD11b+CD11c^lo/neg^Ly6G^hi^Ly6C^int^, and M-MDSCs as CD45 + CD11b+CD11c^lo/neg^Ly6G-Ly6C^hi^. Erythroblasts were classified as previously described [50]. Briefly, erythroblasts (Ter119^hi^) were identified and subdivided based on CD71 expression level and size. In order of increasing maturity, EryA were classified as Ter119^hi^CD71^hi^FSC^hi^, EryB as Ter119^hi^CD71^hi^FSC^lo^, and the most mature EryC as Ter119^hi^CD71^lo^FSC^lo^. Analysis was performed using CytExpert® (Beckman Coulter, Brea, CA, USA).

### Serum analysis

IL-6 and IL-4 levels in mouse serum were measured using the murine IL-6 and IL-4 pre-coated ELISA kit (Peprotech, Rehovot, IL) according to the manufacturer’s instructions.

### Hemoglobin measurement

At the time of sacrifice, mice were bled from the facial vein. The second drop (10 µL) of blood was collected in a capillary tube and subjected to hemoglobin measurement using a hemoglobinometer (Mission® Inc, San Diego, CA, USA).

### Fecal occult blood detection

Feces were collected, weighed, and dissolved in 0.03 M NaOH at 1 mg/mL. In a 96-well plate, 5 μg of feces were placed in 50 μL of luminol working solution (10^-2 ^M luminol, 0.03 M NaOH), followed by the addition of 50 μL 0.03% H_2_O_2_. The plate was read immediately on a luminometer at 425 nm (SpectraMax I3X, San Jose, California, USA). A standard curve was generated by adding blood from a naïve WT mouse with a known concentration of hemoglobin (measured on a hemoglobinometer) to negative control feces from the same naïve WT mouse. Validation of this method is presented in Supplementary Fig. 1.

### Statistical analysis

All analyses for mouse experiments were conducted using GraphPad Prism v9.0. Data were analyzed by Student’s *t*-test, one-way ANOVA, Mann–Whitney *U* test, or Kruskal–Wallis test for continuous variables. Differences in weight loss were analyzed by two-way ANOVA for repeated measures over time. All results are expressed as mean values ± SD unless otherwise indicated. *p* < 0.05 was considered statistically significant.

### Exome-wide association study and gene-based burden test

Exome-wide association study summary statistics and gene-based burden tests, wherein variants are tested in aggregate, for peripheral monocyte count generated by Backman et al. and Barton et al. were accessed via the GWAS Catalog [51, 52]. The monocyte study included 443529 (exome study) or 418449 (gene-based burden test) UKBiobank participants of European ancestry. *CNR2* and its containing haploblock were previously identified [8]. A gene-enrichment analysis of *CNR2* was performed and SNPs strictly in the exomic region with *p* < 0.05 were identified via the Functional Mapping and Annotation (FUMA) platform. The LD matrix for significant and common *CNR2* SNPs was generated using LDlink [53]. Exomic data was validated via the Cohort Browser function on the UKB Research Assistant Platform DNAnexus (ukbiobank.dnanexus.com).

## Results

### OGP has a minimal effect on myelopoiesis and lymphopoiesis in wild-type mice at steady-state

**Fig. 1:**
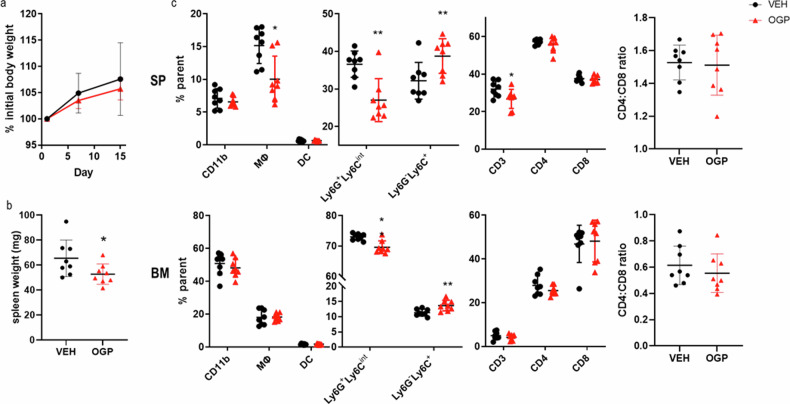
OGP administration in naïve WT mice has a minimal effect on myelopoiesis and lymphopoiesis. **a** Body weight expressed as percent (%) initial of weight recorded on day of first injection (day 0), two-way ANOVA, *p* = 0.4889. **b** spleen weight. **c** Relative frequency of the spleen (SP, top) and bone marrow (BM, bottom) populations of CD11b+ and subpopulations of immature myeloid cells (CD11b+Ly6G^hi^Ly6C^int^ and CD11b + Ly6G-Ly6C^hi^), macrophages (MΦ, CD11b+F480+), dendritic cells (DC, CD11b^hi^ Cd11c^hi^), CD3+, CD3+CD4+, and CD3+CD8+ T cells, and the CD4:CD8 ratio. Indicated values are expressed as percent of the parent population, as determined by flow cytometry analysis. VEH, *n* = 8; OGP (700 ng/week), *n* = 8. Student’s *t*-test, *p < 0.05 and **p < 0.01.

**Fig. 2:**
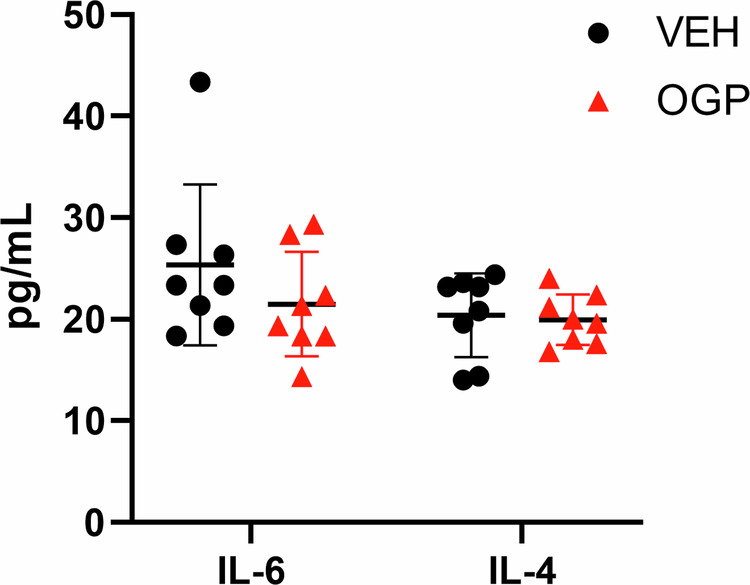
OGP administration in naïve WT mice does not affect IL-6 and IL-4 serum levels. Serum was analyzed by ELISA. Vehicle control (VEH), *n* = 8; OGP (700 ng/week), *n* = 8. Student’s *t*-test.

From previous studies, we observed that OGP is not acutely or chronically toxic in ovariectomy and ear edema models; however, the effect of OGP administration on myelopoiesis and lymphopoiesis at steady-state was not assessed [10]. Due to the role of MDSCs in CRC, and the potential role of OGP therein, we determined the effect of OGP on immature myeloid cells and T cells in the spleen and bone marrow of naïve wild-type (WT) mice. Female mice were injected every seven days with 700 ng of OGP or vehicle control, and sacrificed one day after the third injection. Mice receiving OGP displayed significantly smaller spleens compared to the vehicle control group, although body weight showed no differences (Fig. 1a, b). Flow cytometry analysis revealed a significant decrease in T cells (CD3+) and macrophages in the spleen of OGP-treated mice (Fig. 1c). However, the CD4:CD8 T cell ratio remained unchanged (Fig. 1c). Furthermore, two subsets of CD11b+ immature myeloid cells showed inverse trends as the Ly6G^+^Ly6C^int^ (granulocytic) decreased, while the Ly6G^−^Ly6C^+^ (monocytic) cells increased in both the spleen and bone marrow of OGP-treated mice (Fig. 1c). It is important to note that all values were within the normal range for mice of this age [54–57]. The observed changes in myeloid cell subpopulations did not lead to significant alterations, as the concomitant fluctuations led to an overall neutral effect, corroborated by comparable IL-6 and IL-4 serum levels between groups (Fig. 2).

### OGP attenuates adenoma formation and growth during the progression phase in Apc^Min/+^ mice

**Fig. 3:**
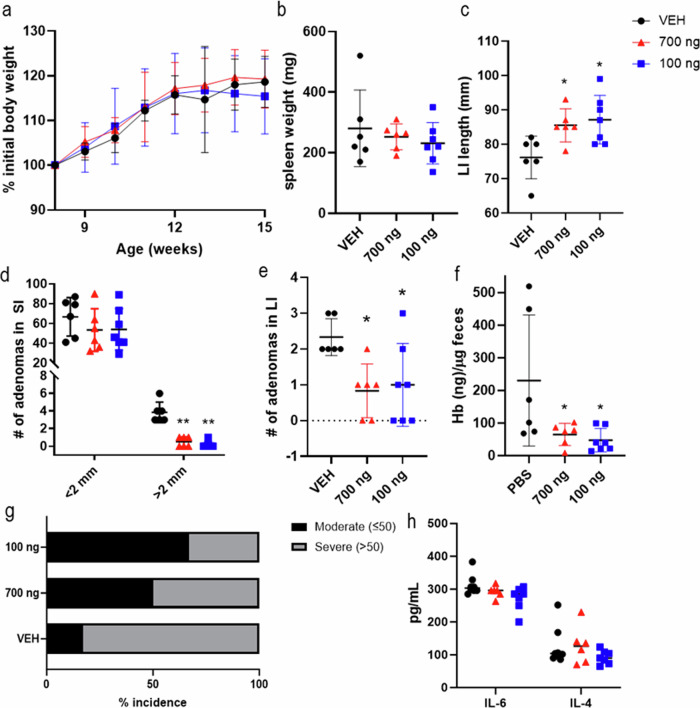
OGP attenuates adenoma formation and growth during the progression phase in Apc^Min/+^ mice. **a** Body weight expressed as percent (%) initial body weight on the day of the first injection, two-way ANOVA, *p* = 0.8389. **b** spleen weight. **c** length of the large intestine. **d** count and size distribution of adenomas in SI. **e** Gross adenomas in the LI (each measuring 2–4 mm). **f** Quantitative analysis of fecal occult blood 1 week before sacrifice. **g** percent incidence of moderate and severe cases based on adenoma count, Fisher’s exact test, *p* = 0.3818 (**h**) IL-6 and IL-4 serum levels determined by ELISA. Vehicle control (VEH), *n* = 6; OGP 700 ng/week, *n* = 6, OGP 100 ng/day, *n* = 7. One-way ANOVA or Kruskal-Wallis test vs VEH, *p < 0.05 and **p < 0.01.

To assess the effect of OGP administration on the progression phase of colon cancer, 8-week-old Apc^Min/+^ mice were injected either once weekly with 700 ng OGP, daily with 100 ng OGP, or vehicle control for 8 weeks and sacrificed two days after the last injection. OGP dosage was determined based on previous experiments using an ear edema model, where a single weekly dose of 700 ng OGP per mouse demonstrated a comparable or even greater effect in reducing ear swelling than a daily dose of 100 ng OGP per mouse (Fig. S1). Mice receiving either dose of OGP showed the same pattern in all parameters and are hereafter described together. Male and female mice were used, as previous studies showed no sex-disparities in cancer development [8]. The body weight and spleen weight showed no difference between groups (Fig. 3a, b). Compared to the vehicle control, OGP-treated mice had significantly milder colonic shortening (Fig. 3c), a sign of severe inflammation and a marker of cancer progression [58]. Although the gross number of adenomas in the small intestine (SI) was not significantly different between groups, OGP-treated mice displayed significantly fewer large adenomas with a diameter greater than 2 mm (Fig. 3d). In the large intestine (LI), there was no difference in size of these adenomas (2-4 mm in all groups) but OGP-treated mice had significantly fewer adenomas than control mice (Fig. 3e). A common CRC screen in humans is the fecal occult blood test to quantify the blood in the stool, as polyps typically bleed intermittently [59]. Using a method we adapted and validated from Park et al. [60] (Fig. S2), we demonstrated that Apc^Min/+^ mice receiving OGP have significantly lower concentrations of blood in the feces compared to the vehicle control group (Fig. 3f). Apc^Min/+^ typically develop at least 50 adenomas throughout their lifespan [28, 61]. We found that 83% of mice treated with vehicle control progressed to a severe condition, as defined by more than 50 adenomas in the small and large intestine combined, compared to 33% (100 ng) and 50% (700 ng) of OGP-treated mice (Fig. 3g). IL-6 and IL-4 serum levels showed no differences between groups (Fig. 3h). Because both CRC patients and Apc^Min/+^ mice suffer from anemia [62, 63], and because CB2 has been implicated in hematopoiesis [64, 65], we asked whether OGP simulates erythropoiesis. We found that administration of OGP for 1-month to 12-week-old naïve WT mice significantly increased hemoglobin in males, but not in females; however, the effect on hemoglobin is no longer present in either sex after 3 months of injections (Fig. S3). In Apc^Min/+^ mice (progression phase), we assessed the relative frequency of erythroblast populations and hemoglobin levels. Flow cytometry analysis of the bone marrow revealed that the subpopulations of erythroid precursors were the same in all groups, corroborated by similar hemoglobin levels (Fig. S4).

### OGP attenuates adenomagenesis during the initiation phase in Apc^Min/+^ mice

**Fig. 4:**
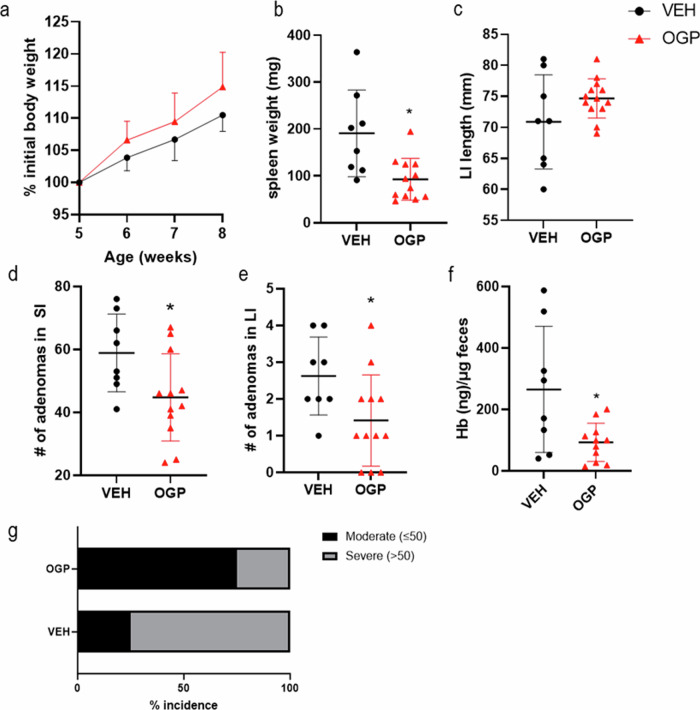
OGP attenuates adenomagenesis during the initiation phase in Apc^Min/+^ mice. **a** Body weight expressed as percent (%) initial body weight on the day of the first injection, two-way ANOVA, *p* = 0.067. **b** Spleen weight. **c** Length of large intestine (LI). **d** Gross adenomas in the small intestine (SI). **e** Gross adenomas in the large intestine (LI, each measuring 2–4 mm). **f** Quantitative analysis of fecal occult blood on the day of sacrifice. **g** Percent incidence of moderate and severe cases based on adenoma count, Fisher’s exact test, *p* = 0.0648. Vehicle control (VEH), *I* = 8; OGP 700 ng/week, *n* = 12. Student’s *t-*test or Mann–Whitney *U* test, *p < 0.05.

To determine the effect of OGP on adenoma formation during the initiation phase, Apc^Min/+^ mice were injected with 700 ng OGP once per week for 4 weeks, starting at 5 weeks of age, and sacrificed 2 days after the last injection. Splenomegaly is a distinct characteristic of Apc^Min/+^ mice [66]. Although the exact etiology is not understood, there is evidence that splenomegaly positively correlates with tumor development in these mice [66]. In this experiment, the difference in body weight was not significant, but OGP-treated mice displayed significantly smaller spleens compared to the vehicle control group (Fig. 4a, b). The OGP-treated mice experienced reduced adenomas in both the SI and the LI, while colon length was the same between groups (Fig. 4c–e). Fecal occult blood levels were significantly lower in OGP-treated mice. (Fig. 4f). Importantly, we found that only 25% of OGP-treated mice progressed to a severe condition (>50 adenomas), compared to 75% of control mice (Fig. 4g).

### OGP reduces MDSC accumulation during the initiation phase in Apc^Min/+^ mice

**Fig. 5:**
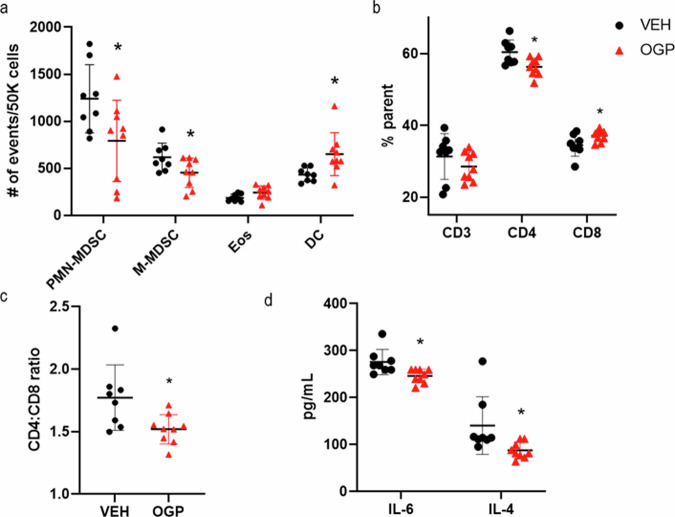
OGP prevents splenic MDSC accumulation during the initiation phase in Apc^Min/+^ mice. **a** Relative frequency of myeloid cells, polymorphonuclear and monocytic MDSCs (PMN- and M-MDSC), eosinophils (Eos), and dendritic cells (DC). **b** Relative frequency of T cells: CD3+, CD3+CD4+, and CD3+CD8+ **c** CD4:CD8 ratio **d** IL-6 and IL-4 levels in the serum. Flow cytometry values are expressed as number of events/50k cells or percent parent as indicated. Vehicle control (VEH), *n* = 8; OGP 700 ng/week, *n* = 9. Student’s *t-*test, *p < 0.05.

As in the progression phase experiment, hemoglobin levels were similar between groups and no significant differences were observed in erythroblast populations of the bone marrow (Fig. S5). OGP treatment in Apc^Min/+^ mice during the initiation phase significantly decreased polymorphonuclear (PMN)- and monocytic (M)-MDSC populations in the spleen (Fig. 5a). Dendritic cells (DCs) were also significantly increased, while eosinophils (Eos) showed no difference compared to the vehicle control (Fig. 5a). Furthermore, OGP treatment altered the balance between CD4 and CD8 T cells in the spleen, with significantly higher CD8+ T cells and significantly lower CD4+ T cells in OGP-treated mice (Fig. 5b, c). This shift in the CD4:CD8 T cell ratio favors a more cytotoxic immune response [67], likely contributing to the anti-tumorigenic effects of OGP in intestinal tumorigenesis. In contrast to the previous experiment started at 8 weeks of age to study tumor progression, the alterations in myeloid and T cell populations were accompanied by a significant decrease in IL-6 and IL-4 serum levels in OGP-treated mice (Fig. 5d).

### OGP diminishes MDSC differentiation and has no apparent effect on colon tumor cells in vitro

**Fig. 6:**
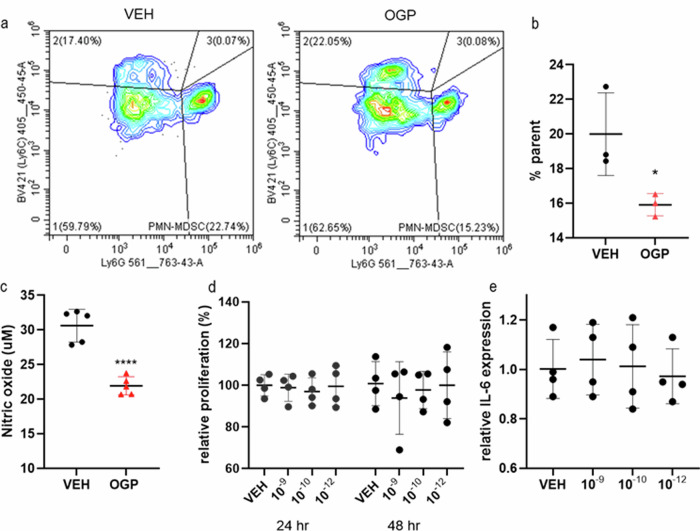
OGP diminishes MDSC differentiation and does not affect CT26 cell proliferation or inflammatory response in vitro. Primary murine bone marrow cells were cultured in CT26-conditioned media and L929-conditioned media to generate MDSCs. **a** Representative flow cytometry plots of PMN-MDSCs (lower right quadrant) frequency in vehicle control (VEH, left) and 10^-12 ^M OGP (right) treated cells grown in media cocktail for 72 h **b** Relative frequency of PMN-MDSCs (Ly6G^hi^Ly6C^int^) expressed as percent of CD45+CD11b+ cells (% parent). Data is representative of three separate differentiation experiments with similar results. **c** Nitric oxide levels in the supernatant of cells grown in media cocktail for 72 h, treated with 10^−12 ^M OGP, and stimulated with 100 ng/mL LPS for 48 h. **d** MTT assay of CT26 cells treated with OGP at the indicated concentrations (M) expressed as relative proliferation compared to VEH. **e** mRNA expression of IL-6 in CT26 cells after OGP treatment for 4 h. n ≥ 3 per group; Student’s *t-*test or One-way ANOVA, *p < 0.05, ****p < 0.0001.

Based on prior studies across multiple cancer models using CB2^−/−^ mice that revealed consistent findings in MDSCs compared to the variable effects observed in T cells [8, 9], coupled with the prominent effect of OGP on the decumulation of MDSCs in Apc^Min/+^ mice, we assessed the effect of OGP on MDSC differentiation using bone marrow-derived cells cultured in the supernatant of CT26 cells—a colorectal carcinoma cell line—to mimic the tumor microenvironment. Bone marrow cells treated with OGP showed a 20% reduction in the differentiation towards the PMN-MDSC phenotype (CD11b+Ly6G^hi^Ly6C^int^) and a decrease in nitric oxide production compared to the vehicle control (Fig. 6a–c). Although subtle differences were seen in M-MDSCs (CD11b+Ly6G^lo/neg^Ly6C^hi^), these differences were not significant and were batch-dependent on CT26-conditioned media. To determine the potential of OGP to act on tumor cells directly, CT26 cells were treated with OGP to assess its effect on proliferation and mRNA expression of IL-6. No apparent differences were seen in proliferation rate as determined via the MTT assay or in IL-6 expression between cells receiving OGP or vehicle control (Fig. 6d, e). Additionally, a quantitative assessment of CB2 surface expression was performed on intestinal epithelial cells from a 16-week-old Apc^Min/+^ mouse and its WT counterpart. We observed that CB2 surface expression on epithelial cells appears to be higher in the WT mouse (Fig. S6a) Due to an immune cell infiltration in the epithelial layer of Apc^Min/+^ mice, we also determined that CB2 was expressed on 24% of immune cells (CD45 + ) and 20% of myeloid cells (CD11b + ) (Fig. S6b, c).

### Association of common and rare CNR2 variants and peripheral blood monocyte count in humans

**Fig. 7:**
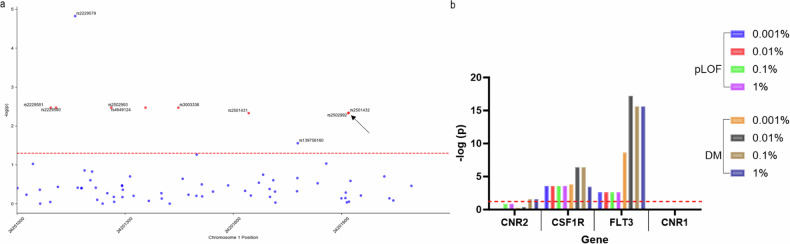
Association of rare variants of *CNR2* with anal polyp occurrences and common and rare variants with monocyte count. **a** Manhattan plot of p values in the exomic region of CNR2 (build GRCh37) showing common variants (red, MAF > 1%) and rare variants (blue, MAF ≤ 1%) associated with monocyte count. Q63R variants are indicated with a black arrow. **b** Gene-based burden test of rare variants of CNR2, genes associated with monocyte proliferation (*CSF1R* and *FLT3*), and *CNR1* for putative loss of function mutations (pLOF) and putative loss of function deleterious missense mutations (DM) at the indicated allele frequencies.

Due to the dichotomous effect seen in granulocytic and monocytic immature myeloid cells in the steady-state murine experiment, coupled with a previously established link between CNR2 variants and lymphocyte count and eosinophil count [38, 39], we perfromed an additional analysis of the association of peripheral blood monocyte count and CNR2 variants in UKBiobank participants, using previously generated exomic association study summary statistics [52]. In the present analysis, we found that many common variants as well as the two SNPs contributing to the functional variant Q63R (rs2502992 and rs2501432) are significantly associated with a lower monocyte count (*p* < 0.05, Fig. 6a). These polymorphism are in strong linkage disequilibrium (LD, *r*^2^ = 1) with the other common variants with an allele frequency >10% (Fig. S7). An assessment of the gene-based burden test generated by Backman et al. [51] showed that rare CNR2 variants (MAF < 1% and <0.1%) that cause a deleterious missense mutation were also significantly associated with monocyte count (Fig. 7b). The effect of the rare variants is far more moderate than the effect of genes directly involved in monocyte survival and proliferation—colony-stimulating factor 1 receptor (CSF1R), and fms-like tyrosine kinase 3 (FLT3)—although still notably significant compared to CNR1 [68, 69].

## Discussion

The intricate pathophysiology of colon cancer encompasses a complex interplay of genetic predispositions and dynamic interactions among various immune cell populations, contributing to the onset and progression of the disease. The results of our study demonstrate the immunomodulatory and anti-tumorigenic role of CB2 activation via its endogenous selective agonist OGP in both the initiation and progression phases of colon cancer. Our findings highlight the context-dependent nature of OGP, as evidenced by its distinct behavior under cancer-associated inflammation and steady-state conditions, in addition to its established anti-inflammatory effect in models of acute inflammation [10].

In naïve mice, we observed OGP treatment had a minimal overall effect on myelopoiesis, indicating this CB2 agonist has no immunosuppressive actions in healthy animals. Although the subpopulations of immature myeloid cells we tested (Fig. 1) are similar to MDSCs and have the same surface markers, MDSCs are not present at steady-state [70]. The accumulation of MDSCs is a multifaceted phenomenon and hinges on two signal types: the first signal prompts the proliferation of immature myeloid cells while inhibiting their final maturation, and the second signal drives the pathological activation of these cells, transforming them into MDSCs [70]. While the effect of OGP was physiologically negligible in naïve mice, the potential of this peptide to impact proliferation of immature myeloid cells, *i.e*. the first signal, was still observed, indicating that these cells are a principal target of OGP. By comparing naïve WT mice to Apc^Min/+^ mice, we show this effect of OGP is biologically significant in cancer in that MDSCs impact adenoma growth and formation, but insignificant in the absence of inflammation.

Previous studies indicated that exogenous administration of OGP induces the secretion of endogenous OGP in a positive feedback loop [71]. It was also previously demonstrated that OGP(1-14) forms complexes with circulating binding proteins to protect from proteolysis, providing a sustained-release phenomenon [71, 72]. Because of these unique characteristics, along with the subtle influence of OGP seen in the steady-state experiment, we assessed two low doses of OGP, 700 ng once weekly and 100 ng daily, to determine if one weekly injection is sufficient in mitigating adenomagenesis. Indeed, we found that during the progression phase, wherein adenoma growth is the predominant feature, both OGP doses exhibited the same anti-tumorigenic effect with significantly smaller adenomas in the SI, and decreased incidence in the LI. In a previous study on the initiation phase of CRC, we reported that Apc^Min/+^ mice lacking the CB2 receptor (Apc^Min/+^CB2^−/−^) have more prominent adenomagenesis [8]. In the absence of endogenous CB2 activation, pro-tumor cells in the tumor microenvironment and spleen, namely PMN-MDSCs and M-MDSCs, are more abundant [8]. As such, we observed the inverse effect upon OGP administration. In the initiation phase of CRC, wherein adenomagenesis is rampant, OGP treatment evidently reduced adenoma formation. This effect was corroborated by a prominent depletion in the splenic populations of PMN-MDSCs, M-MDSCs, and CD4+ T cells, along with decreased serum levels of IL-6 and IL-4, cytokines known to promote proliferation of MDSCs and tumor growth [20, 23]. This prevailing evidence supports the notion that OGP mediates MDSC proliferation and/or IL-6 secretion, thus inhibiting adenomagenesis. Differences in IL-6 and IL-4 were not seen in the progression phase. Whereas this finding might be attributed to extensive inflammation evident in later stages of cancer in which the effect of OGP is more moderate on cytokine secretion or immune cell proliferation, it prompts a consideration of CB2 activation on intestinal epithelial cells, as their expression of CB2 in more advanced phases of cancer is increased [73].

Apc^Min/+^ mice lose weight gradually after 14 weeks of age, perhaps explaining why no differences in weight loss were observed in either phase, although a decrease in spleen weight was noted in mice receiving OGP treatment during the initiation phase [74]. Likewise, anemia increases substantially in severity around 6 months of age, with reports showing normal hemoglobin levels and no red blood cell abnormalities in young mice [30, 75]. The occurrence of anemia can be partially attributed to the presence of bleeding polyps, resulting in a notable presence of blood in the stool [76]. Although we found significant differences in fecal occult blood in both phases of cancer, we did not observe significant differences in hemoglobin levels or erythroid precursors between groups, with all mice showing the recognized marked shift towards immature erythropoiesis. We found that male WT mice treated with OGP for 1 month do not experience age-related hemoglobin reduction, although this effect is not present in males or females after 3 months of OGP administration. Other reports suggest that OGP has the potential to impact hematopoietic regeneration; however, there is also evidence that CB2 activation via endogenous ligands has no major effects on immature hematopoiesis in a model of hematopoietic stress [12, 64]. While we observed myelopoiesis to be significantly altered in OGP-treated mice in our colon cancer model, differences in erythropoiesis were not seen, perhaps due to an age-dependent mechanism or biased signaling and functional selectivity of CB2 activation, alluding to the context-dependent nature of OGP.

Previous reports have suggested the anti- or pro-tumorigenic effect of CB2 stems from its direct activation on immune cells in the tumor microenvironment, peripheral immune cells, or tumor cells [77–79]. As we observed a prominent decumulation of MDSCs in Apc^Min/+^ mice treated with OGP, we performed a series of in vitro experiments on primary murine myeloid cells grown in conditioned media to mimic the tumor microenvironment to determine if OGP inhibits the differentiation towards the MDSC phenotype. Notably, we found that OGP treatment diminishes the PMN-MDSC subpopulation. These OGP-treated cells also secreted less nitric oxide, a well-established effect of CB2 agonists in macrophages [80]. Coupled with our data showing that OGP does not effect CT26 proliferation of IL-6 expression (a cytokine essential for CT26 tumor survival in vivo), we found that OGP has the potential to act directly on myeloid cells. While it is quite possible that OGP is acting on epithelial cells, we observed that CB2 expression is lower on epithelial cells in the colons of Apc^Min/+^ mice compared to WT, further supporting a direct effect of OGP on immune cells, particularly myeloid cells.

As we previously found common *CNR2* variants are associated with colon cancer in humans and data from mice experiments shows a role of CB2 in immune-cell modulation, we evaluated common and rare variants associated with monocyte count. We showed a likely connection between monocyte count in peripheral blood and rare and common *CNR2* variants. For the common variants, the mutation increases the likelihood of a lower monocyte count. This aligns with the effect of OGP in naïve mice on monocytic immature myeloid cells in the spleen and bone marrow. Upon activation of CB2 by OGP, this population of cells significantly increased, whereas variants—particularly those contributing to Q63R leading to a less functional CB2 [81]—conceivably decrease monocyte count in the peripheral blood. Specific cell population data, further than a standard complete blood count, is not available from the UKBiobank; as such, we speculate if CNR2 functionality could affect specific monocyte subtypes more than others, affecting the balance between pro- and anti-inflammatory monocytes.

Overall, our results portray OGP as a potent anti-tumorigenic peptide, particularly in the initial stages of colon cancer, with significant immunomodulatory functions. Our findings provide valuable insights into the multifaceted role of OGP, thereby establishing a foundation for future therapeutic strategies employing OGP in oncological interventions.

## Supplementary information

Supplementary Material

## Data Availability

Data were generated by the authors unless otherwise stated and are available upon request. Genomic data are publicly available through the GWAS catalog.
