# Supplementary material for: Immunomodulatory function of cannabinoid receptor 2 and its agonist osteogenic growth peptide in health and cancer: a study in mice and humans
Source: Oncogene. 2025 Apr 30;44(29):2504–14. doi: 10.1038/s41388-025-03399-9 (PMC12256265; doi:10.1038/s41388-025-03399-9)
Supplement: Supplementary file 1 — Supplementary Material [file 41388_2025_3399_MOESM1_ESM.pdf]

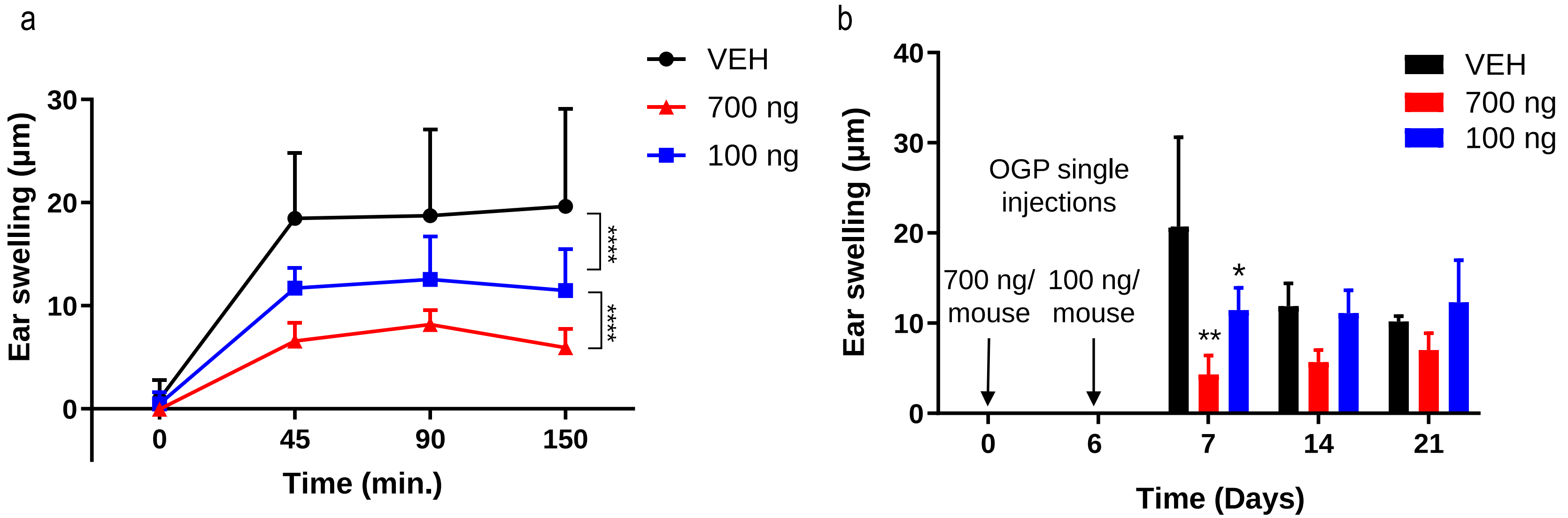

Fig S1 The **e**ffect of OGP on xylene-induced skin acute inflammation (external ear). **a** Single injection of OGP 700 ng/mouse or 100 ng/mouse were injected 7 days or one day before the first xylene application, respectively and compared to the vehicle control (VEH). Ear thickness measurements were performed before the injection and after 45 min, 1.5 h, and 2.5 h. using a Mitutoyo calliper that applies a standard pressure on the tissue. Data are shown as mean ± SD, n=6 mice per group. Two-way ANOVA statistical test versus the OGP 100 ng group, **** P value < 0.0001. **b** Single injection of OGP 700ng/mouse or 100ng/mouse, injected 7 days or one day before the first xylene application, respectively as shown in the graphs (Day 0, 700 ng OGP injection and on day 6, 100 ng OGP). Xylene application and ear thickness measurements were performed on days 7, 14 and 21. Data are shown as mean ± SD for the 2.5h time point only (results at day 7 are the same as in a at 2.5 hrs only), 3<n<6 mice per group. Two-way ANOVA statistical test. vs. Vehicle-treated group for each time point. * P value < 0.05, ** P value < 0.001.

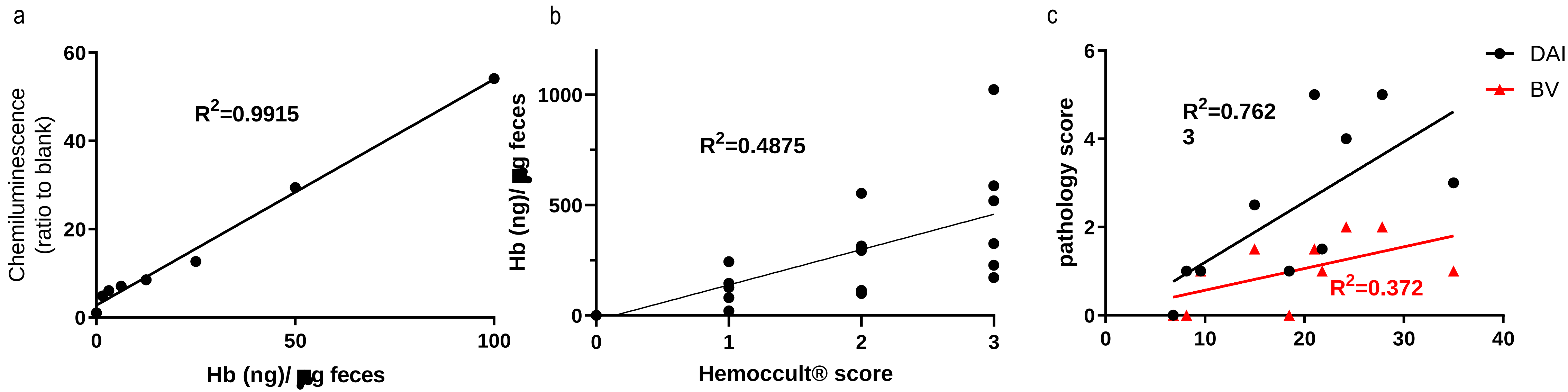

Fig. S2 Validation of fecal occult blood detection via the luminol reaction method. Fecal occult blood in experimental mice was determined based on the concentration of hemoglobin measured using the luminol reaction and determined using curve. **a** standard curve generated using blood taken from naïve mice with a known hemoglobin (Hb) value as measured by a hemoglobinometer and distributed at the indicated concentrations onto feces (from the same naïve mice) dissolved in a luminol solution. A reaction solution containing hydrogen peroxide was added immediately before reading on a luminometer. **b** Comparison of developed method to Hemoccult® kit using the feces from Apc$^{Min/+}$ mice of varying disease progression. Fecal occult blood scores were determined using the Hemoccult® kit, a guaiac-based assessment, and blindly given a score from 0-3 based according to the manufacturer's instructions. Feces from the same mice and timepoint were analyzed via the luminol reaction method. **c** Relationship between disease activity index (DAI) and regularity of blood vessel organization on the mucous membrane (BV) pathology scores as measured blindly by colonoscopy in mice receiving AOM/DSS treatment (chemical induction of colon cancer) and fecal occult blood measured using the luminol reaction method at the time of colonoscopy

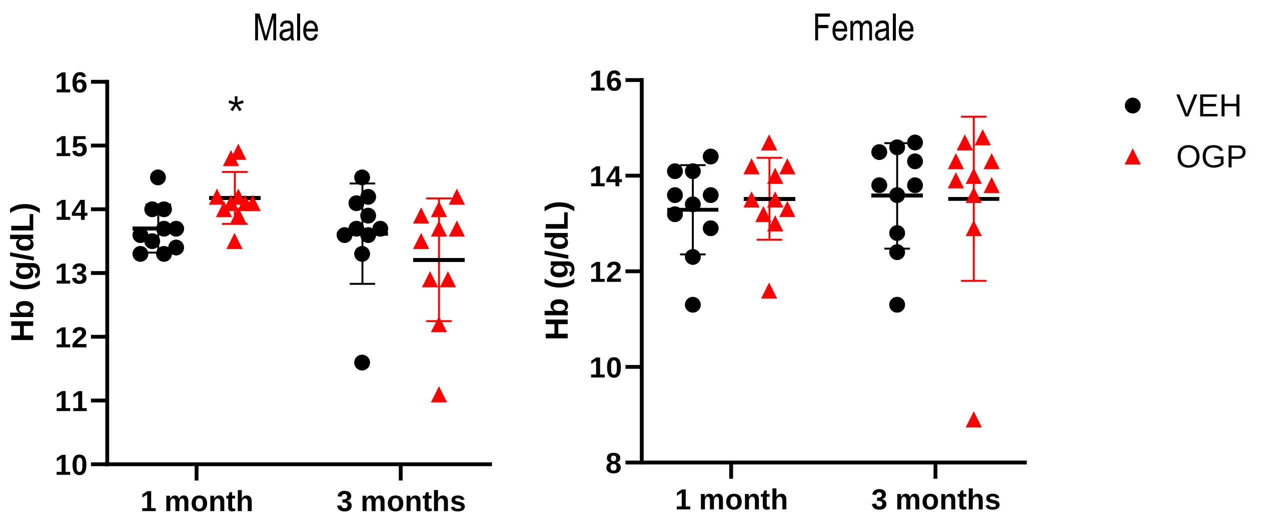

Fig. S3 The effect of OGP on hemoglobin (Hb) in wildtype male and female mice. Starting at 12 weeks of age, mice were injected weekly with 700 ng OGP for one month or three months. Vehicle control (VEH), n= 10. OGP (700 ng/week), n=10. Student's t test. * $p<0.05$ vs. VEH

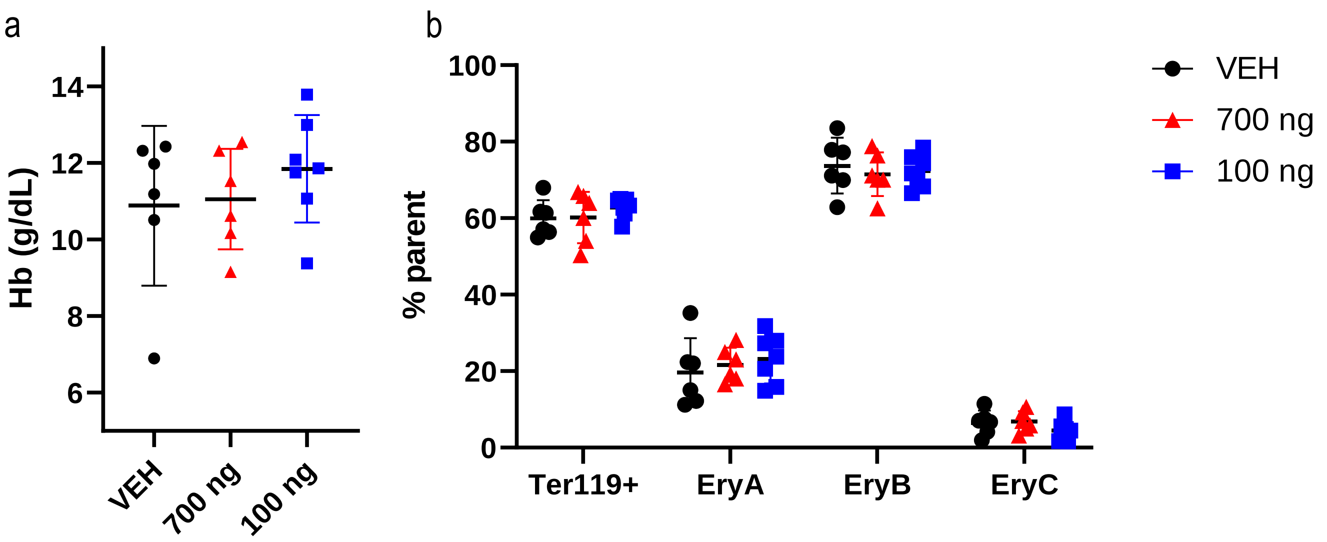

Fig. S4 The effect of OGP on hemoglobin and erythroblasts in $Apc^{Min/+}$ mice during the progression phase. Mice were injected with 700 ng/week or 100 ng/day or vehicle control for four weeks, starting at 5 weeks of age. **a** Hemoglobin (Hb) levels. **b** relative frequency of erythroblasts (Ter119+), $Ter119^{hi}CD71^{hi}FSC^{hi}$ (EryA), $Ter119^{hi}CD71^{hi}FSC^{lo}$ (EryB), and ($Ter119^{hi}CD71^{lo}FSC^{lo}$(EryC). Vehicle control (VEH), n=6; OGP 700 ng/week, n=6, OGP 100 ng/day, n=7. One-way ANOVA vs VEH

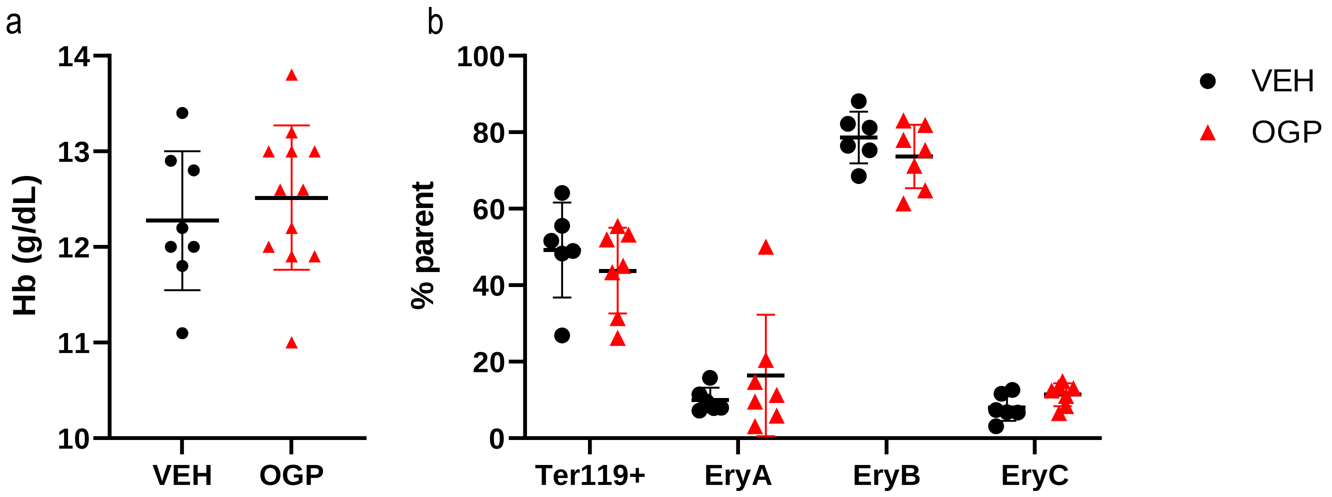

Fig. S5 The effect of OGP on hemoglobin (Hb) and erythroblasts in $Apc^{Min/+}$ mice during the initiation phase. Mice were injected weekly with 700 ng OGP for eight weeks, starting at eight weeks of age. **a** Hemoglobin (Hb) levels. **b** relative frequency of erythroblasts (Ter119+), $Ter119^{hi}CD71^{hi}FSC^{hi}$ (EryA), $Ter119^{hi}CD71^{hi}FSC^{lo}$ (EryB), and ($Ter119^{hi}CD71^{lo}FSC^{lo}$(EryC). Vehicle control (VEH), n≥7; OGP 700 ng/week, n ≥7. Student's *t*-test

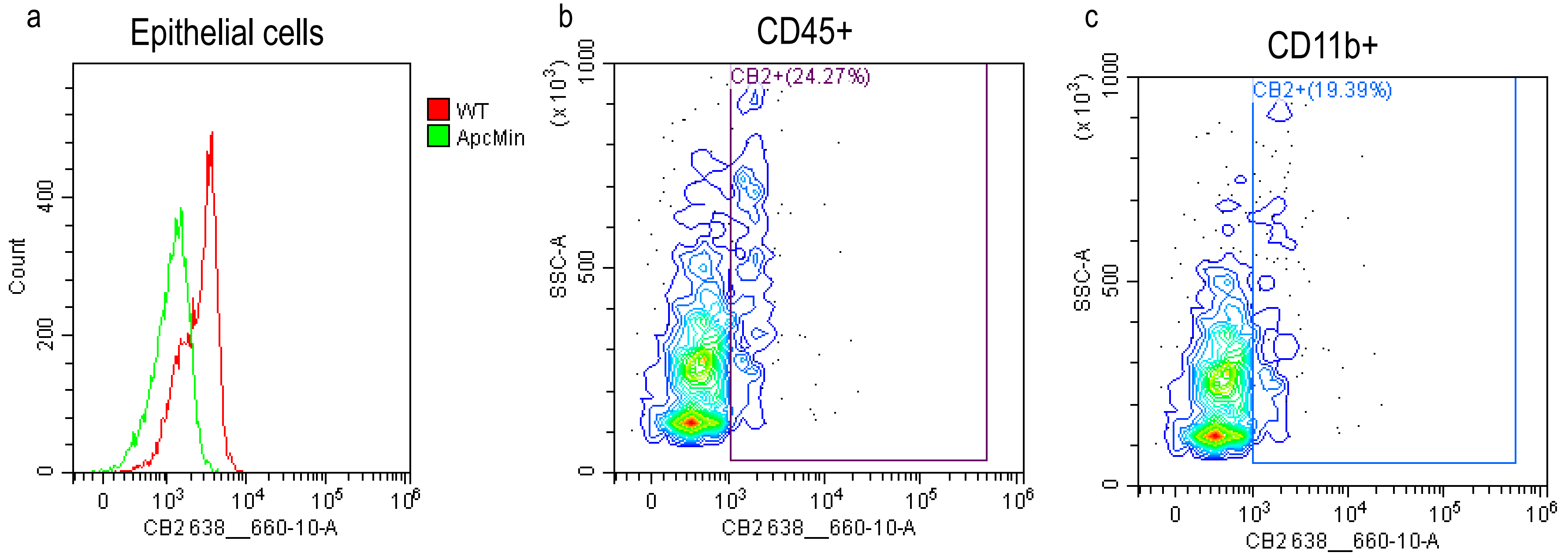

Fig. S6 Quantitative assessment of CB2 expression in epithelial and immune cells in the large intestine of $Apc^{Min/+}$ mice. **a** histogram overlay demonstrating the level of CB2 surface expression on colon epithelial cells (EPCAM+) in WT (red) compared to $Apc^{Min/+}$ (green). CB2 surface expression in **b** immune cells (CD45+) and **c** myeloid cells (CD11b+) in $Apc^{Min/+}$ mice.

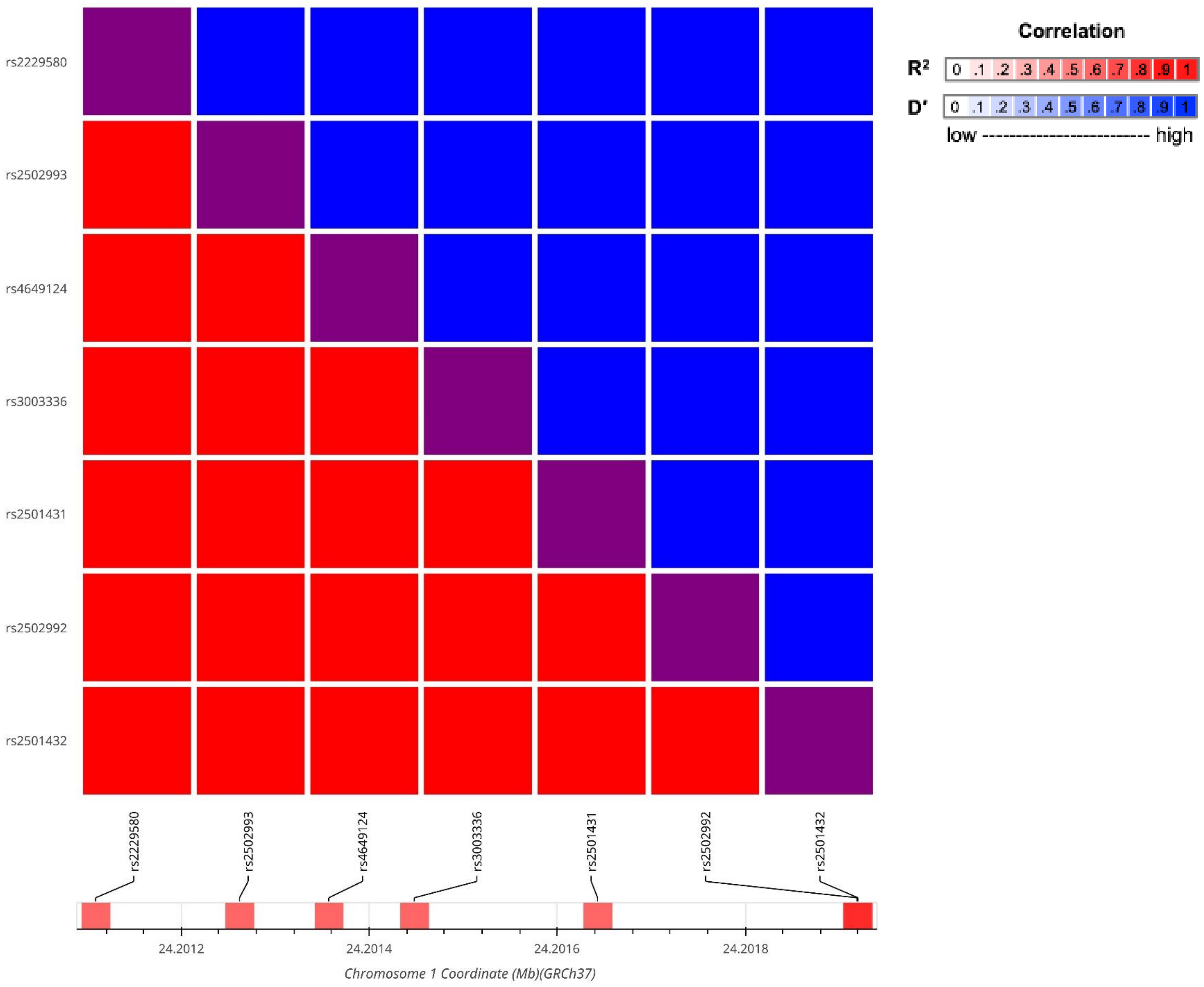

Fig. S7 LD matrix of *CNR2* biallelic variants in the exomic region with minor allele frequency >10% significantly associated with monocyte count
